# pRb-mediated control of epithelial cell proliferation and Indian Hedgehog expression in mouse intestinal development

**DOI:** 10.1186/1471-213X-7-6

**Published:** 2007-01-26

**Authors:** Hai-Su Yang, Philip W Hinds

**Affiliations:** 1 Molecular Oncology Research Institute, Tufts-New England Medical Center, 75 Kneeland Street, Boston, MA 02111, USA

## Abstract

**Background:**

Self-renewal of the epithelium of the small intestine is a highly regulated process involving cell proliferation and differentiation of stem cells or progenitor cells located at the bottom of the crypt, ending ultimately with extrusion of the terminally differentiated cells at the tip of villus.

**Results:**

Here, we utilized the Cre/*lox*P system to investigate the function of the retinoblastoma protein, pRb in intestinal epithelium. pRb null mice displayed a profoundly altered development of the intestine with increased proliferation and abnormal expression of differentiation markers. Loss of pRb induces cell hyperproliferation in the proliferative region (crypt) as well as in the differentiated zone (villi). The absence of pRb further results in an increase in the population of enterocytes, goblet, enteroendocrine and Paneth cells. In addition, differentiated enteroendocrine cells failed to exit the cell cycle in the absence of pRb. These proliferative changes were accompanied by increased expression of Indian hedgehog and activation of hedgehog signals, a known pathway for intestinal epithelial cell proliferation.

**Conclusion:**

Our studies have revealed a unique function of pRb in intestine development which is critical for controlling not only the proliferation of a stem cell or progenitor cell population but that of terminally differentiated cells as well.

## Background

The functional epithelium of the mammalian adult small intestine is organized into finger-like villi which project into the gut lumen and flask-like crypts which are embedded in the mesenchyme [1]. The mammalian intestinal epithelium is characterized by continuous self-renewal and differentiation along the distinct crypt-villus axis. In the crypt region, stem cells and progenitor cells proliferate and give rise to the four main differentiated cell types: the enterocytes, the goblet cells, the entero-endocrine cells and the Paneth cells [2]. Tissue homeostasis in the gut epithelium involves multiple processes like cell proliferation, differentiation, migration, adhesion, and cell lineage allocation [1]. This highly dynamic system appears particularly well suited for investigating key biological phenomena such as the relationship between execution of a terminal differentiation program and cell cycle regulation.

The retinoblastoma protein (pRb) is a prototype tumor suppressor and plays a crucial role in cell cycle regulation, operative in all cell types. A universal function for Rb in tumor suppression is implied by the frequent inactivation of the cell cycle regulatory pathway centered on Rb in most human cancers [3,4]. Challenging a potentially ubiquitous role for Rb in development, however, is the fact that embryos nullizygous for Rb alone, or in combination with other family members, show cell lineage-specific defects [5,6]. Mice lacking pRb die in utero displaying defects in erythropoiesis, the CNS and retina [7-9]. Whereas mice with null mutations in either *p107 *or *p130 *are viable and normal, mice lacking both *p107 *and *p130 *die at birth with defects in endochondral bone development associated with inappropriate cell cycle exit [10,11]. This indicates that p107 and p130 perform overlapping functions that cannot be carried out by pRb. Similarly, pRb may have overlapping functions with p130 that are not shared with p107 [12]. Mice deficient for both pRb and p107 show an aggravated *Rb*-null phenotype [11] , Finally, mouse embryo fibroblasts lacking all three Rb family members are immortal and do not respond to senescence inducing signals [13,14]. Furthermore, the Rb family has been implicated in several aspects of differentiation processes such as terminal cell cycle exit, maintenance of the post-mitotic state and induction of tissue-specific gene expression [6].

The embryonic lethality of *Rb*-null mutant mice by 14 days of gestation (E14) precludes the phenotypic analysis of pRb in the development of the intestine, since intestinal villi begin to form at E14.5 [15]. At that time, the intestinal lumen is surrounded by a multilayered stratified epithelium. During the next 24 hr, in proximal-to-distal fashion, the intestinal lining undergoes a dramatic morphological change, resulting in the formation of abundant villi. During villus formation, the stratified epithelium is converted to a single layer of columnar epithelium as the underlying mesenchyme condenses and evaginates to form the villus core. By E16.5 the process is complete and mitosis becomes restricted to the intervillus epithelium, which matures postnatally to become the stem cell-containing crypts of the adult intestine [15]. Studies have found that in adult intestine, pRb is an important determinant of proliferative status during terminal differentiation of the enterocytic lineage. This terminal differentiation is associated with loss of cyclin D1 and cdk2 but *not *their partners, cdk4 and cyclin E [16]. Therefore, understanding how expression of these cell cycle regulators is controlled along the crypt-villus axis should provide more profound insights into the mechanisms that permit the continuum between proliferation and differentiation (and death) to be established and maintained in this dynamic, self-renewing system.

Here, we employed the Cre/*lox*P system to study the function of pRb in the development of the mouse intestine. Mice lacking RB displayed an abnormal intestinal phenotype with increased proliferation and aberrant numbers of differentiated cells. Loss of RB stimulated the generation of intestinal cells apparently by upregulating Indian hedgehog and its downstream targets, suggesting that pRb plays a critical role in controlling immature proliferating progenitor cells and terminal cell differentiation.

## Results

### Cre-mediated RB ablation results in an abnormal development of intestine

Intestine is a muscular hose-like portion of the gastrointestinal tract extending from the lower end of the stomach (pylorus) to small intestine and then to the anal opening (large intestine). Small intestine is composed of duodenum, jejunum, and ileum [2]. The processes of proliferation and differentiation are coordinately regulated in the intestinal epithelium. To identify the function of pRb in the development of intestine, we utilized a conditional knockout approach based on the Cre/*lox*P system [17]. The *lox*P RB mice were created with *lox*P sites flanking exon 19 of the RB gene and RB can be ablated via Cre-mediated excision [18]. The Cre-recombinase mice were generated with Cre under the control of collagen1A1 promoter (3.6 Col1A-Cre) [19]. The offspring of RB^wt/f19^, 3.6 Col1A-Cre expressing transgenic line (RB^+/-^) were bred with RB^f19/f19 ^strain (RB^+/+^). We observed that RB^f19/f19^, Col1A1-Cre mice (RB^-/-^) were dead at birth and were of smaller size than RB heterozygous littermates.

**Figure 1:**
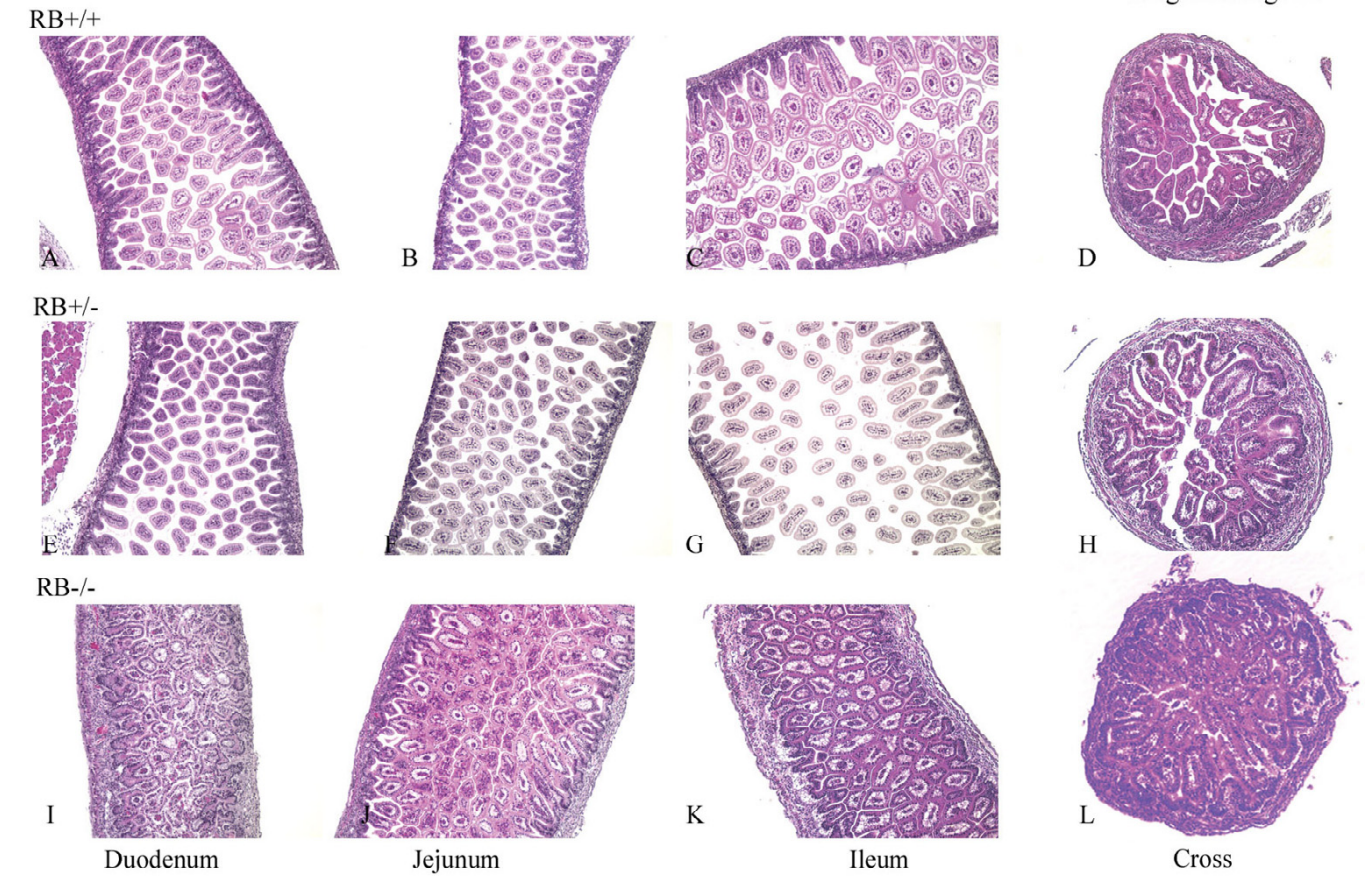
**Cre-mediated RB ablation results in abnormal development of small intestine**. Haematoxylyn-Eosin (H/E) staining was performed on intestine sections including duodenum (A, E, I), jejunum (B, F, J) and ileum (C, G, K) as well as cross sections (D, H, L) derived from RB^+/+ ^(A, B, C, D), RB^+/- ^(E, F, G, H) and RB^-/- ^(I, J, K, L) newborn mice.

To delineate the role of pRb in embryonic intestinal development, we carefully monitored the effect of pRb loss on formation of gut in newborn pups. Histological analysis showed that the intestines in duodenum, jejunum, and ileum from RB^+/+^, RB^+/- ^mice displayed a well-developed structure and density of villi (Fig. 1A–H). In contrast, the intestinal tracts of the RB^-/- ^mice revealed a distinctly different phenotype: Villi became blunt and fused. The lamina propria underlying the villi exhibited increased thickness, and the central blood vessels within the villi were less prominent than in the control littermates. Mucosal architecture from the duodenum to the ileum was severely distorted with highly increased villus density in RB^-/- ^mice. The high amount of villi in RB^-/- ^pups filled in the whole lumen area (Fig. 1I–L). These results strongly indicate that pRb is essential for normal intestinal development. Loss of pRb induces apparent hyperproliferation of intestinal cells resulting in abnormal gut formation.

To determine whether Cre was expressed in intestine at a level allowing recombination, PCR analysis was performed to detect Cre expression in DNA extracted from intestinal tissues of E17.5 day embryos. Cre expression was readily detected by PCR analysis and correlated with loss of RB exon19 in intestine from RB^+/- ^and RB^-/- ^[see Additional file 1]. The recombination occurred in RB heterozygous and null mice, with retention of 680 bp fragment representing the intact locus of wild-type RB only in RB^+/- ^mice [see Additional file 1].

Total cell lysates isolated from the intestine of E17.5 day embryos were used to analyze the protein expression of pRb by immunoblotting. Upon Cre expression and recombination, the removal by Cre of exon 19 of the RB gene resulted in loss of RB expression in intestine from E17.5 embryos. Whereas intestines derived from RB^+/- ^mice expressed normal levels of pRb, RB null intestine showed complete loss of pRb expression [see Additional file 1]. In addition, lysates from RB deficient intestines displayed an increase in p107 levels [see Additional file 1], similar to that seen pRb-null fibroblasts [13,14,20]. However, we observed no changes in p130 expression among the different genotypes of RB [see Additional file 1].

### Loss of pRb induces the proliferation of intestinal cells

**Figure 2:**
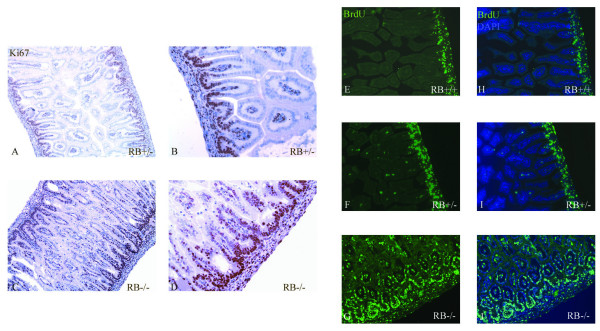
**Disruption of pRb induces cell proliferation**. The embryonic intestine sections (E17.5) derived from RB^+/- ^(A, B) or RB^-/- ^(C, D) mice were stained by IHC with proliferating cell marker Ki67. The number of proliferative cell in RB null intestine was highly increased. An enlargement of the portion in the left-hand micrograph (A, C) is shown in the right-hand (B, D) micrograph. BrdU positive cells in embryonic intestine sections derived from RB^+/+ ^(E, H), RB^+/- ^(F, I) and RB^-/- ^(G, J) mice were stained with anti-BrdU antibody conjugated with Alexa Fluor 488 (E, F, G) or DAPI (H, I, J).

In the adult intestine epithelial cells are constantly renewed through migration and differentiation along the crypt-villus axis from a small number of actively dividing stem cells present in mucosal invaginations at the villus base (crypts). The increase in density of villi in RB^-/- ^mice suggests a defect in the regulation of proliferation and/or differentiation. To address this, we measured proliferation antigen ki67 in intestinal sections obtained from RB^+/- ^and RB^-/- ^E17.5 day embryos. As illustrated in Figure 2, proliferative cells in the intestine of RB^+/- ^embryos were concentrated in a distinct proliferative zone in the crypt region at the base of the villi (Fig. 2A, B). However, RB null embryos showed an increased number of ki67 positive cells that localized not only in the crypt area but also throughout the villus (Fig. 2C, D). To further test whether the absence of RB results in an ectopic S-phase entry, BrdU incorporation assay was performed. In intestines derived from RB wt and heterozygous newborn pups, BrdU positive cells were only localized at the bottom of crypt region (Fig. 2E, F, H, I), whereas BrdU positive cells extended from the crypt to the tip of villi in RB null mice (Fig. 2G, J). These data indicate that pRb is required for cell cycle exit in intestinal cells. Loss of RB resulted in persistent S phase and increased cell numbers in gut. In addition, no significant apoptosis was observed in villi from RB^+/+^, RB^+/- ^and RB^-/- ^new born pups as revealed by staining for active caspase-3 [see Additional file 2], although dead cells did appear on the tip of the villi in 3-week of normal adult mice [see Additional file 2], suggesting that loss of pRb itself did not lead to alterations in rates of cell death in embryonic intestine development. These results demonstrate that pRb plays a pivotal role in establishing and/or maintaining the intestinal villus compartment.

### Lack of RB stimulates the expression of intestinal differentiated cells

**Figure 3:**
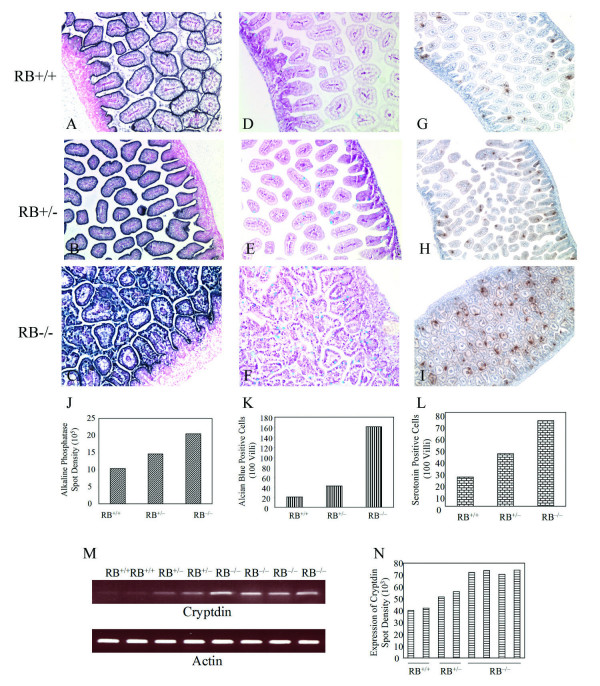
**Lack of RB stimulates enterocytes, goblet, enteroendocrine and Paneth cells**. Representative sections of duodenum from RB^+/+ ^(A, D, G), RB^+/- ^(B, E, H) and RB^-/- ^(C, F, I) newborn pups were stained with Alkaline phosphatase (A, B, C), Alcian blue (D, E, F) and IHC using anti-serotonin antibody (G, H, I). Nuclear fast red was used as a counterstain for AP and Alcian blue staining. The quantified activity of Alkaline phosphatase was measured by the density of image (J). Alcian blue positive cells (K) or serotonin positive cells (L) were counted in 100 villi derived from RB^+/+^, RB^+/- ^and RB^-/- ^intestines. Small intestine from newborn pups was subjected to RT-PCR using primers specific to cryptdin-1 and actin as a control (M). The amount of cryptdin-1 was evaluated through the density of signal (N).

Studies have found that all four epithelial cell lineages of the intestine are differentiated from common pluripotent stem cells in the crypt region of the intestine [2,21]. However, the mechanisms that regulate this epithelial cell differentiation are not fully defined. Using specific differentiation markers, we characterized the function of pRb in cell fate determination in intestinal development. An alkaline phosphatase activity assay showed that more dark purple staining of enterocytes occurred at the brush border of the intestine in RB^+/- ^newborn pups than in RB^+/+ ^littermates, and enterocytes in RB^-/- ^mice displayed the strongest alkaline phosphatase activity (Fig. 3A–C, & quantified in Fig. 3J). Similarly, alcian blue positive goblet cells increased to the highest amount in RB null pups compared to RB heterozygous and wild type mice, while loss of one RB allele in RB^+/- ^pups slightly increased goblet cell numbers compared to that in RB^+/+ ^mice (Fig. 3D–F, K). The enteroendocrine cell lineage in the intestine was also analyzed. The specific endocrine marker serotonin was strongly detectable in RB^+/- ^pups compared to the amount of serotonin positive cells in RB wild type mice. Again, RB null pups showed the highest abundance of serotonin positive cells in the intestine (Fig. 3G–I, L). Paneth cells are granulated cells that do not become mature until the first 2 neonatal weeks. However, cryptdin-1, one of the earliest markers in Paneth cells, has been detected as early as E15.5 [22,23]. Cryptdin-1 expression levels were analyzed by RT-PCR amplification of intestinal RNA from P0 mice. The amount of cryptdin-1 in intestine from RB wild type pups was low, while RB^+/- ^pups showed a slight increase in expression of cryptdin-1. In contrast, RB null mice had the highest amount of cryptdin-1 expression (Fig. 3M, N). These results indicate that loss of pRb stimulates persistent cell cycling but allows cell differentiation in the intestine. These data demonstrate that pRb must be tightly regulated to allow orderly control of cell proliferation and cell lineage specification along the crypt-villus axis.

### Differentiated enteroendocrine cells remain in the cell cycle in the absence of pRb

**Figure 4:**
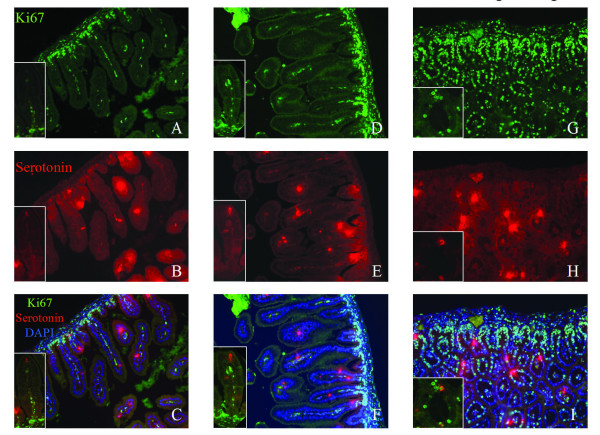
**Differentiated enteroendocrine cells have an ability to remain in the cell cycle due to loss of pRb**. Intestinal sections from RB^+/+ ^(A, B, C), RB^+/- ^(D, E, F) and RB^-/- ^(G, H, I) newborn mice were stained by immunofluoresence by using anti-Ki67 (Green) (A, D, G) and anti-serotonin (Red) (B, E, H). Merged images with anti-Ki67 (Green), anti-serotonin (Red) and DAPI (Blue) are shown at the bottom (C, F, I).

As shown in Figure 2, loss of pRb in the intestine results in more proliferating cells as assessed by staining for the cell proliferation marker, Ki67. We further wanted to determine whether differentiated cells in villi from pRb null mice have the ability to proliferate. Double staining for Ki67 and serotonin to identify enteroendocrine cells was performed in intestine sections from RB^+/+^, RB^+/- ^and RB^-/- ^newborn pups. Clearly no serotonin positive cells colocalized with Ki67 positive proliferating cells which were only detected at the base of villi in intestines from RB heterozygous and wt mice (Fig. 4C, F). In contrast, staining for ki67 (nucleus) and serotonin (cytosol) colocalized in many intestinal epithelial cells in the RB null mice. 35% of serotonin-positive cells co-stained for Ki67, while the remaining Ki67 positive cells were separated from differentiated serotonin positive cells (65%) (Fig. 4I), indicating that the differentiated enteroendocrine cells had the capability to remain in the cell cycle in the absence of pRb.

### Loss of pRb upregulates the expression of Indian hedgehog

**Figure 5:**
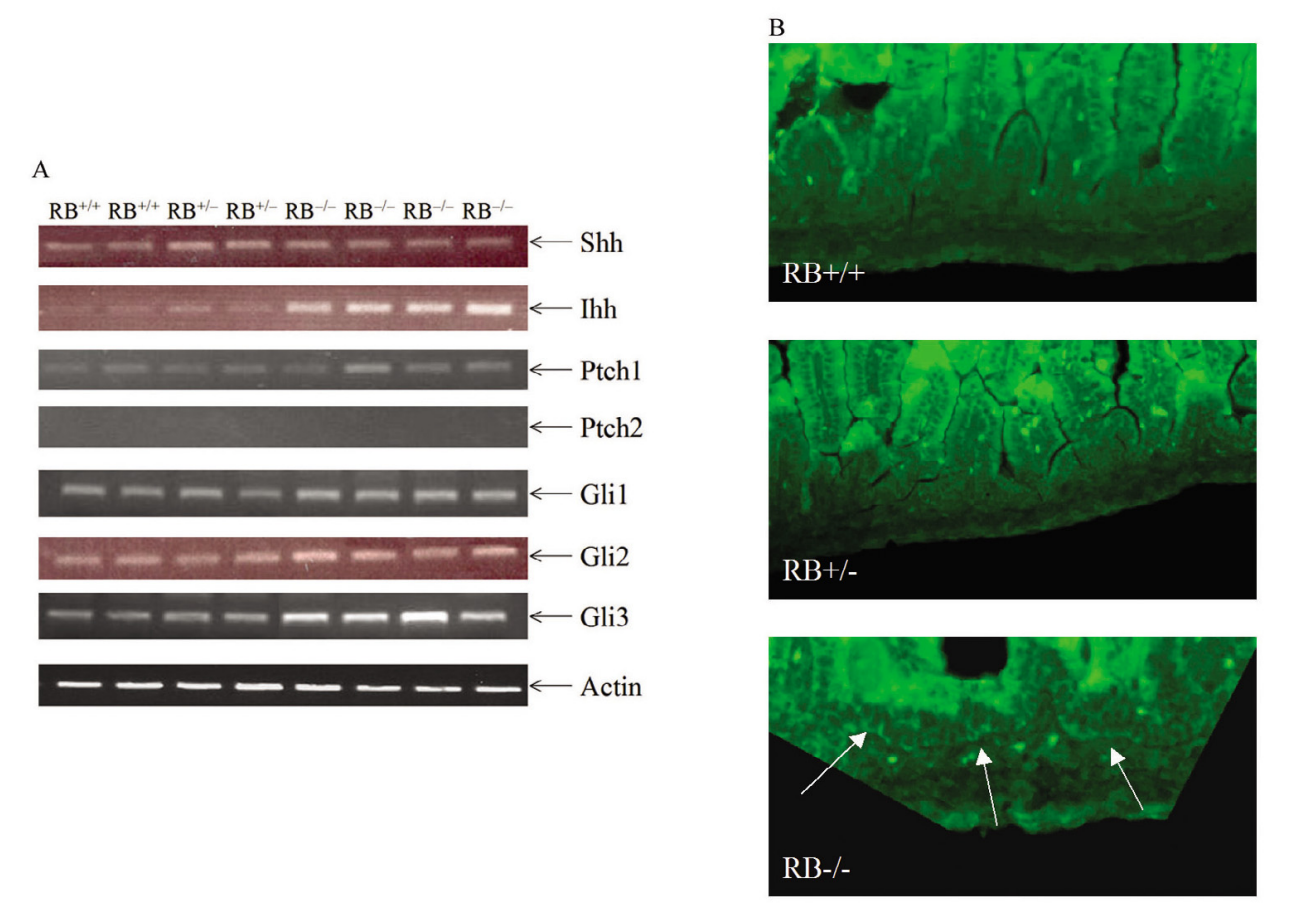
**Loss of pRb stimulates Indian hedgehog expression**. (A) RT-PCR from P0 intestines derived from RB^+/+^, RB^+/- ^and RB^-/- ^newborn pups were performed to detect the expression levels of Shh, Ihh, Ptch1, Ptch2, Gli1, Gli2, Gli3 and actin used as a control. (B) Immunofluoresence analysis of Ihh in intestine sections derived from RB^+/+^, RB^+/- ^and RB^-/- ^newborn pups was performed by using anti-Ihh antibody. Arrows indicate regions of increased anti-Ihh immunostaining.

Previous studies have found that hedgehog (Hh) signals regulate multiple aspects of gastrointestinal development [24]. Indian hedgehog (Ihh) and Sonic hedgehog (Shh) are the two murine homologues of the *Drosophila *Hh protein. The response to Hh ligands is mediated by two transmembrane proteins, Smoothened (Smo) and Patched (Ptc), and by downstream transcription factors of the Gli family [25]. In the absence of Hh, Ptc suppresses the signaling activity of Smo. When Hh binds to Ptc, Ptc is functionally inactivated, enabling Smo to signal [25]. Here we analyzed the effect of pRb on the expression of hedgehog members. Quantitative RT-PCR amplification revealed that Indian hedgehog (Ihh) was highly induced in intestines derived from RB null newborn pups, although sonic hedgehog (Shh) expression was little changed (Fig. 5A). Only ptch1, not ptch2 mRNA was found in intestine derived from RB^+/+^, RB^+/- ^and RB^-/- ^newborn pups. Moreover, the expression levels of Gli transcription factors were analyzed. Loss of RB upregulated the expression of Gli3 while the levels of Gli1 or Gli2 had no apparent alteration (Fig. 5A). The localization of Ihh was further determined by immunofluorescence by using anti-Ihh antibody. In RB^-/- ^newborn pups, Ihh staining was highly increased in the subepithelial region at the base of the villi which is considered stem cell compartment location, whereas Ihh staining was hardly detectable in RB^+/+ ^and RB^+/- ^pups (Fig. 5B). These results strongly suggest that pRb plays an important role in intestinal stem cells and/or progenitor cells. pRb may function to control hedgehog protein expression and downstream signals to regulate the proliferation/differentiation of progenitor cells. Loss of RB may stimulate Indian hedgehog expression and its signaling which then contributes to the observed intestinal abnormal proliferation and differentiation of intestinal epithelium, consistent with previous studies that demonstrate that Ihh is required to maintain the intestinal stem-cell compartment [24].

## Discussion

The lethality at mid-gestation due to germ line loss of RB in mice precludes the analysis of pRb deficiency at later stages of development. The use of conditional knockout approaches based on the Cre/*lox*P system allows analysis of the tissue-specific and temporal requirements for pRb function during development and in the adult animal without the complications associated with germ line gene inactivation. Previous work has established that expression of SV40 large T antigen, which disrupts E2F-Rb complexes, leads to the reentry of intestinal enterocytes into the cell cycle and intestinal hyperplasia [26] , indicating a potential role for the Rb family proteins in mediating intestinal homeostasis. However, we are still far from understanding how pRb might regulate stem cell or progenitor cell proliferation and terminal differentiation in the intestine. Here, our data indicate that 3.6 Col1A1-Cre can efficiently cause the recombination and inactivation of "floxed" RB alleles in the embryonic intestine. Loss of pRb results in abnormal intestinal development associated with defects in proliferation and differentiation. The increased proliferation in pRb-null villi leads to increased number of differentiated cells. Four types of intestinal cells, enterocytes, goblet cells, enteroendocrine cells and Paneth cells are highly induced in RB null mice. Furthermore, inactivation of RB appears to stimulate the expression of Indian hedgehog and its signal pathway, which might contribute to the proliferation and differentiation of intestinal cells.

Differentiated epithelial cells in the intestine are believed to originate from multipotent stem cells located in the crypt region [1]. These stem cells slowly duplicate and give rise to a transient population of progenitor cells that rapidly divide. Most types of intestinal epithelial cells move upward toward the gut lumen as they mature while Paneth cells migrate downward to the crypt region. During their migration, precursor progenitors in the small intestine become committed towards one of four different lineages: enterocytes, goblet cells, enteroendocrine cells and Paneth cells. These cells undergo cell cycle arrest and functional differentiation as they leave the crypt and migrate to the villus or to the bottom of the crypt (Paneth cells) [1]. However, the mechanisms controlling cell division, cell fate specification and migration along the crypt-villus axis remain incompletely understood.

The retinoblastoma protein (pRb) is at the heart of the cellular machinery that controls passage from G1 into S phase of the cell cycle. It is also critical for many aspects of differentiation [10,27,28]. Thus, we suspected that pRb might affect the proliferation potential of intestinal cells and that pRb could serve to control the number of stem cells and progenitor cells located at the crypt region. Indeed, our results suggest that the absence of RB stimulates cell proliferation (Ki67 and BrdU) not only at the crypt region but throughout entire of the villi as well (Fig. 2). Intriguingly, the proliferative cells colocalized with differentiated cells in villi (at least as shown for enteroendocrine cells) (Fig. 4), suggesting differentiated intestinal cells had an ability to remain in the cell cycle in the absence of pRb. These results suggest that pRb could mediate the proliferation control in stem cells or progenitor cells as well as in "fully" differentiated cells, since all differentiated cell types were persistent in high numbers in pRb-null intestines. However, rigorous identification of stem cell and progenitor cell in the intestine remains difficult due to a lack of definitive morphological criteria or molecular markers. Thus, it is impossible at this time to determine with certainty whether our transgenic model does indeed affect intestinal stem cells or early precursor cells as well as differentiated cells. The use of intestinal epithelial cell specific Cre transgene, such as villin Cre [29] , is ongoing in our lab and may help to define the compartment(s) sensitive to pRb loss in the intestinal epithelium.

To begin to understand the molecular nature of the abnormal proliferation and differentiation of pRb-null intestinal epithelium, we examined multiple signal pathways that are involved in the regulation of intestinal development. Notch signaling plays an important role in cell specification in the intestine. Mice deficient for hairy and enhancer of split (HES-1), a transcriptional repressor downstream of Notch signaling, results in an increased secretory cell population at the expense of adsorptive cells [30]. Hes-1 represses Math1 expression in intestine [30] , suggesting that the absorptive versus secretory fate decision is likely to be established through the Notch-Hes1 pathway. However, in our system, mice deficient for pRb have no significant effect on the expression of Notch1, Notch3, Hes1 and Hes5 (not detectable) [see Additional file 3], nor on Math1, Neurogenin3 (not detectable), and NeuroD expression [see Additional file 3], indicating that the cell lineage decision in intestine derived from RB^+/+ ^or RB^-/- ^mice is probably not altered through Notch and basic helix loop helix transcription factor signaling.

In addition, the Wnt pathway has been found to be important for stem cell proliferation and/or lineage differentiation in the intestinal epithelium. Loss of TCF-4, a downstream effector of the Wnt cascade, results in accumulation of cell cycle arrested, differentiated cells [31] , indicating the important function of the β-catenin/TCF pathway in the development of intestine. Intriguingly, we found no change in this pathway, including Wnt1 (not detectable), Wnt2 (not detectable), Frizzled, Frizzled3 (not detectable), Dickkopf1 (DKK1, Wnt antagonist) (not detectable) in intestine derived from RB^+/+^, RB^+/-^and RB^-/- ^newborn pups [see Additional file 4]. Further, loss of pRb in the intestine affected neither TCF-4 nor LEF1 (not detectable) nor the nuclear localization of β-catenin in intestinal cells [see Additional file 4]. These observations suggest that Wnt signaling/Notch signaling probably is not involved in alterations observed in pRb-null intestines.

In contrast to the observed lack of alteration in compounds of Notch and Wnt signaling, loss of RB stimulated the expression levels of Indian hedgehog which was concentrated in the intervilli region at the base of the villi in RB newborn pups. Ihh expression in epithelial cells is required for stem cell proliferation and commitment of cell lineages. Loss of Ihh in transgenic mice results in reduced epithelial stem cell proliferation and differentiation [24]. Because our data revealed an increase in all differentiated compartments including enterocytes, goblet cells, enteroendocrine cells and likely Paneth cells in intestines derived from RB deficient mice, a role for factors like Ihh that stimulate early progenitor cell proliferation seems likely. Furthermore, our data indicated that the expression level of Gli3 transcriptional factor, the downstream targets of hedgehog signal pathway, was stimulated in RB null mice, suggesting that pRb might play a critical role in the regulation of hedgehog signaling to control intestinal cell proliferation and differentiation. Studies have shown that Hh signaling has a distinct ability to promote proliferation by upregulating cyclin D and cyclin E [32] , which act in part by inducing phosphorylation and inactivation of pRb. These results thus indicate that pRb and Ihh hedgehog may function in a feedback loop to control the proliferation of intestinal cells. pRb may repress Ihh, resulting in decreased cyclin expression to tightly control cell proliferation in normal intestine development. However, there is no apparent E2F binding site located at the promoter region of Indian hedgehog, indicating that pRb probably modulates the expression of Ihh through E2F-independent pattern or other transcriptional factors. The study about how pRb regulates Ihh at transcriptional level is ongoing in our lab. On the other hand, the presence of pRb could also modulate the expression of differentiation-related genes by itself or through hedgehog signaling in immature progenitor cells and prevent premature differentiation of other epithelial lineages in the proliferating crypt region until these cells further differentiate and enter the villus compartment. Indeed, the expression levels of Cdx1 and Cdx2, which play an important role in intestinal development, have been reported to be elevated in another model of pRb loss in the intestine when combined with p130 loss [33] , and such deregulation of Cdx1 and Cdx2 also have seen in our pRb-null intestines (data not shown). However, in the previous studies, changes in intestinal epithelial cells proliferation were much more limited than in our study, and no alteration in Ihh was reported, suggesting that the target cell and/or timing of pRb loss can have marked effects on phenotype.

All together, it is clear that cell growth and proliferation must be carefully regulated and coordinated with the processes of cell patterning and differentiation in intestinal development. pRb clearly has an important role in coordinating these processes, but at present, it is unclear whether the effects of pRb loss are solely due to deregulation of Ihh expression and changes in the stem or progenitor cell compartment or if cell autonomous changes in cell cycle exit or differentiation specific gene expression also contribute. Interestingly, during the preparation of this manuscript, it was reported that intestinal epithelial cells, specific loss of pRb caused only limited hyperproliferation of a subset of differentiated intestinal cells [34] , underscoring the much more profound phenotypic consequence of pRb loss in progenitor cells in our system. In any case, it is clear that understanding the regulation of intestinal cell proliferation by pRb will provide significant insight into the mechanisms of cell lineage specification and stem/progenitor cell function in the intestine.

## Conclusion

Our work has demonstrated a novel ability of pRb to regulate intestinal development. Mice lacking pRb as a result of conditional knockout based on the Cre/*lox*P system display a defect in development of the intestine with increased proliferation and expression of differentiation markers. In addition, loss of RB stimulated the generation of intestinal cells by upregulating Indian hedgehog and its signal pathway. These studies suggest a unique function of pRb in intestinal development which is critical for controlling not only the proliferation of a stem cell or progenitor cell population, but cell lineage terminal differentiation as well.

## Methods

### Transgeic mice

RB^f19/f19 ^[18] and Collagen1A1-cre mice (3.6 Col-cre) mice [19] are kindly provided by Dr. Anton Berns and Dr. Barbara Kream respectively. Tail DNA or intestine DNA was isolated with lysis buffer (50 mM Tris-HCl, pH 8.0, 100 mM EDTA pH 8.0, 100 mM NaCl, 1% SDS) and freshly added proteinase K at 0.5 mg/ml. Mice were genotyped by PCR analysis using primers Cre 5' GAGTGATGAGGTTCGCAAGA 3' and 5' CTACACCAGAGACGGAAATC 3'; flox19 5' AACTCAAGGGAGACCTG 3' and 5' GGCGTGTGCCATCAATG 3'.

### Histological Analysis

Embryonic intestine was dissected from mice immediately after euthanasia of the animals. Intestine tissues were fixed in 4% paraformaldehyde in PBS overnight at 4°C, embedded in paraffin, cut into 5-μm sections, and stained with hematoxylin and eosin (H&E). For alkaline phosphatase staining, tissues were deparaffinized, incubated with AP buffer (100 mM Tris-HCl, pH9.5, 100 mM NaCl, 5 mM MgCl2, 005% Tween 20), and stained with BCIP/NBT substrate (Sigma). For Alcian blue staining, sections were incubated with 3% acetic acid containing 1% Alcian blue (pH2.5). The slides were placed in 0.1% nuclear fast red as a counterstain.

### Immunoblot

Total proteins were extracted from freshly dissected intestine by homogenizer with lysis buffer (50 mM Hepes, pH7.5, 150 mM NaCl, 1 mM EDTA, 0.1% NP-40) and freshly added protease and phosphatase inhibitors (50 mM NaF, 1 mM Na_3_VO_4_, 1 mM PMSF, 1 mM benzamidine, 1 mM DTT, 25 μg/ml aprotinin, 25 μg/ml trypsin inhibitor, 25 μg/ml leupeptin, 2 mM β-glycerophosphate). Cell lysates were heated for 5 min. at 95°C in 2× sample buffer (100 mM Tris, pH6.8, 2% SDS, 20% glycerol, 0.04% bromophenol blue and 2% β-mercaptoethanol). Proteins were separated by SDS-PAGE and electroblotted onto Hybond ECL nitrocellulose membranes (Amersham). After blocking with 5% non-fat dried milk in TTBS buffer (20 mM Tris, pH7.5, 500 mM NaCl, 0.05% Tween-20), the blots were incubated with primary antibodies against pRb (BD Pharmingen), p107 (AbCam) and p130 (AbCam) and glyceraldehyde-3-phosphate dehydrogenease (GAPDH) (Chemicon International). The blots were incubated with peroxidase conjugated AffiniPure goat anti-mouse IgG (Jackson ImmunoResearch Laboratories, Inc.) and visualized by ECL detection (Amersham).

### RT-PCR analysis

Total RNA was isolated from freshly dissected intestine with Trizol reagent (Invitrogen). Complementary DNA synthesis was performed according to manufacturer's instructions (SuperScript kit; Invitrogen). For PCR amplification reactions, the primers used were as follows: mouse cryptdin-1, 5' AAGAGACTAAAACTGAGGAGCAGC 3' and 5' GGTGATCATCAGA CCCCAGCATCAGT 3'; Shh 5' CGGCCGATATGAAGGGAAGA 3' and 5' CGGAGTTCTCTGCTTTCACA 3; Ihh 5' GGCCATCACTCAGAGGAGTC 3' and 5' CCGAATGCTCAGACTTGACA 3'; Patched1 5' TGTCTGGCATCAGTGAGGAG 3' and 5' GACAAGGAGCCAGAGTCCAG 3'; Patched2 5' CCTCCGCACCTCATATCCTA 3' and 5' TTGGTGTAGTGCAGCTCCTG 3'; Gli1 5' CTCGACCTGCAAACCGTAAT 3' and 5' GTGGTACACAGGGCTGGAGT 3'; Gli2 5' ACCATGCCTACCCAACTCAG 3' and 5' TCATCCCTGTCCAGGTCTTC 3'; Gli3 5' ATGGACAGCACCAGATTTCC 3' and 5' GGAACCACTTGCTGAAGAGC 3'; Math1, 5' AGATCTACATCAACGCTCTGTC 3' and 5' ACTGGCCT CATCAGAGTCACTG 3'; Ngn3, 5' CTTCACAAGAAGTCTGAGAACACCAG 3' and 5' CTGCGCATAGCGGACCACAGCTTC 3'; NeuroD, 5' GCATGCACGGGCTGAACGC 3' and 5' GGGATGCACCGGGAAGGAAG 3'; Notch1, 5' CGGTGAACAATGTGGATGCT 3' and 5' ACTTTGGCAGTCTCATAGCT 3'; Notch3, 5' GAGGCTACCTTGGCTCTGCT 3' and 5' GGCAGCCTGTCCAAGTGATCT 3'; Hes1, 5' CAGCCAGTGTCAACACGACAC 3' and 5' TCGTTCATGCACTCGCTGAG 3'; Hes5, 5' AAGTACCGTGGCGGTGGAGATGC 3' and 5' CGCTGGAAGTGGTAAAGCAGCTT 3'; TCF-4, 5' CGAGATATCAACGAGGCTTTCAAG 3' and 5' CATGTGATTCGCTGCGTCTCC 3'; LEF1, 5' CTCAACACGAACAGAGAAAGG AGCAGG 3' and 5' GTACCTGAAGTCGACTCCTGTAG3'; β-actin, 5' GACGGCCAGGTC ATCACTAT 3' and 5' ACATCTGCTGGAAGGTGGAC 3'.

### Brdu Incorporation

Animals were injected with 200 μg/g of 5-bromo-2-deoxyuridine (BrdU) 1 hour prior to sacrifice. Embryonic intestinal sections were deparaffinized and trypsinized with 0.1% trypsin for antigen retrieval. BrdU positive cells were recognized with anti-BrdU antibody conjugated with Alexa Fluor 488 (1:100 dilution, Molecular Probes) by immunofluorescence.

### Immunohistochemistry and immunofluorescence

For immunohistochemistry, tissues were deparaffinized through the xylene and ethanol procedure, and quenched with hydrogen peroxide. Antigen retrieval was achieved by boiling in 10 mM citrate buffer pH 6.0. Slides were then subjected to immunohistochemistry with the following primary antibodies: anti-Ki67 (1:100 dilution; BD Pharmingen), anti-cleaved caspase-3 (1:200 dilution; Cell Signalling), anti-serotonin-1 (1:10000 dilution, ImmunoStar). Slides were washed in PBS and incubated with biotinylated secondary antibody (Vector Laboratories). Sections were then rinsed with PBS, incubated with ABC reagent (Vector Laboratories), and developed with the diaminobenzidine peroxidase (DAB) substrate kit (Vector Laboratories). For immunofluorescence, sections were incubated with PBS containing 5% BSA, followed by primary antibodies anti-Ki67 (1:100 dilution; BD Pharmingen), anti-serotonin-1 (1:1000 dilution, ImmunoStar), anti-Ihh (1:250 dilution, Santa Cruz). Slides were washed with PBS and incubated with fluorophore-conjugated secondary antibody. After staining, coverslips were mounted using Fluoromount G.

## Authors' contributions

HSY participated in the design of the study, executed the experiments, collected and organized the data, and wrote the manuscript. PWH provided advice on the study design throughout the project, helped interpret data, and edited the manuscript.

## Supplementary Material

Additional file 1 Collegen1A1-specific Cre expression leads to pRb excision in vivo. (A) Cre-mediated recombination of "floxed" RB was detected by PCR analysis on intestine DNA isolated from E17.5 embryos (left). Cre transgene expression was assessed by PCR (right) (B) The expression levels of pRb family members: pRb, p107, and p130 in intestine derived from E17.5 embryos were detected by immunoblot analysis. GAPDH was used as a control. Click here for file

Additional file 2 Ablation of pRb has no effect on apoptosis. Apoptosis was detected with an antibody that recognizes cleaved caspase-3. Paraffin embedded intestine sections were derived from 3-week adult mice (A) and RB^+/+^, RB^+/- ^and RB^-/- ^newborn pups (B). Several apoptotic cells were detected on the tip of villi in adult mice, whereas different genotypes of RB newborn mice do not show apoptotic figures at this developmental stage. Click here for file

Additional file 3 Notch/Math pathway has little effect on the abnormal intestine with loss of pRb. RT-PCR amplification from P0 intestines derived from RB^+/+^, RB^+/- ^and RB^-/- ^newborn pups were performed to detect the expression levels of Delta1, Delta3, Jagged1, Jagged2, Notch1, Notch3, Hes1, Hes3 and actin used as a control (A) as well as Math1, Neurogenin3, and NeuroD (B). Click here for file

Additional file 4 Wnt signal does not contribute to the defect of intestine in the absence of pRb. Intestines derived from RB^+/+^, RB^+/- ^and RB^-/- ^newborn pups were subjected to RT-PCR amplification for the detection of Wnt1, Wnt2, Frizzled1, Frizzled3, DKK and actin used as a control (A) as well as TCF-4 and LEF1 (D). The embryonic intestine sections (E17.5) derived from RB^+/- ^(left) or RB^-/- ^(right) mice were stained by IHC with β-catenin (B). Representative sections from RB^+/+^, RB^+/- ^and RB^-/- ^newborn pups were stained with immunofluoresence by using anti- β-catenin (Green) and DAPI (Blue) (C). Click here for file
