# Supplementary figures and images for: pRb-mediated control of epithelial cell proliferation and Indian Hedgehog expression in mouse intestinal development
Source: BMC Dev Biol. 2007 Jan 26;7:6. doi: 10.1186/1471-213X-7-6 (PMC1794236; doi:10.1186/1471-213X-7-6)

A

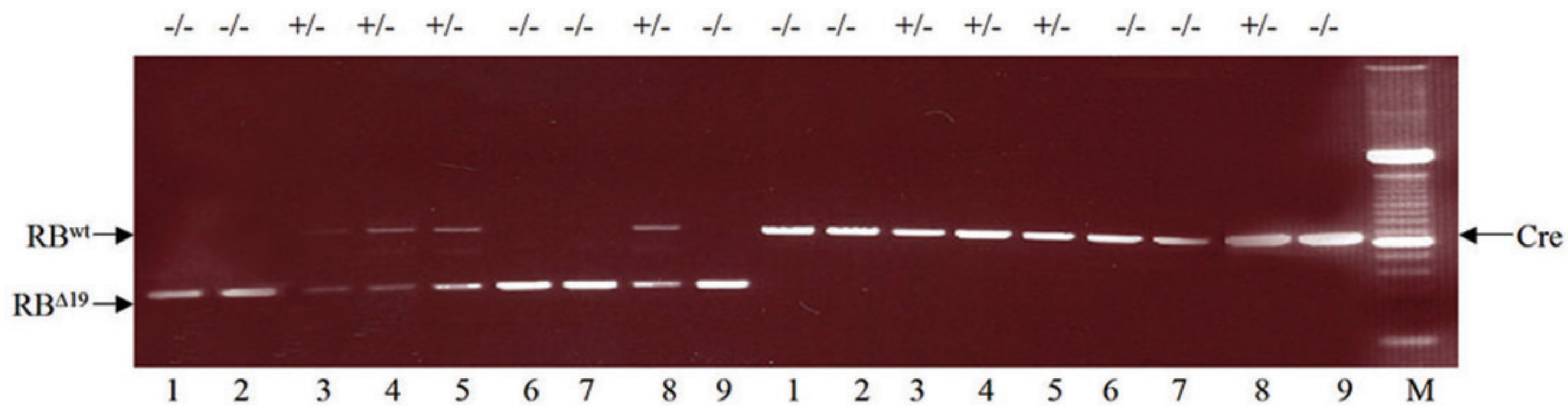

B

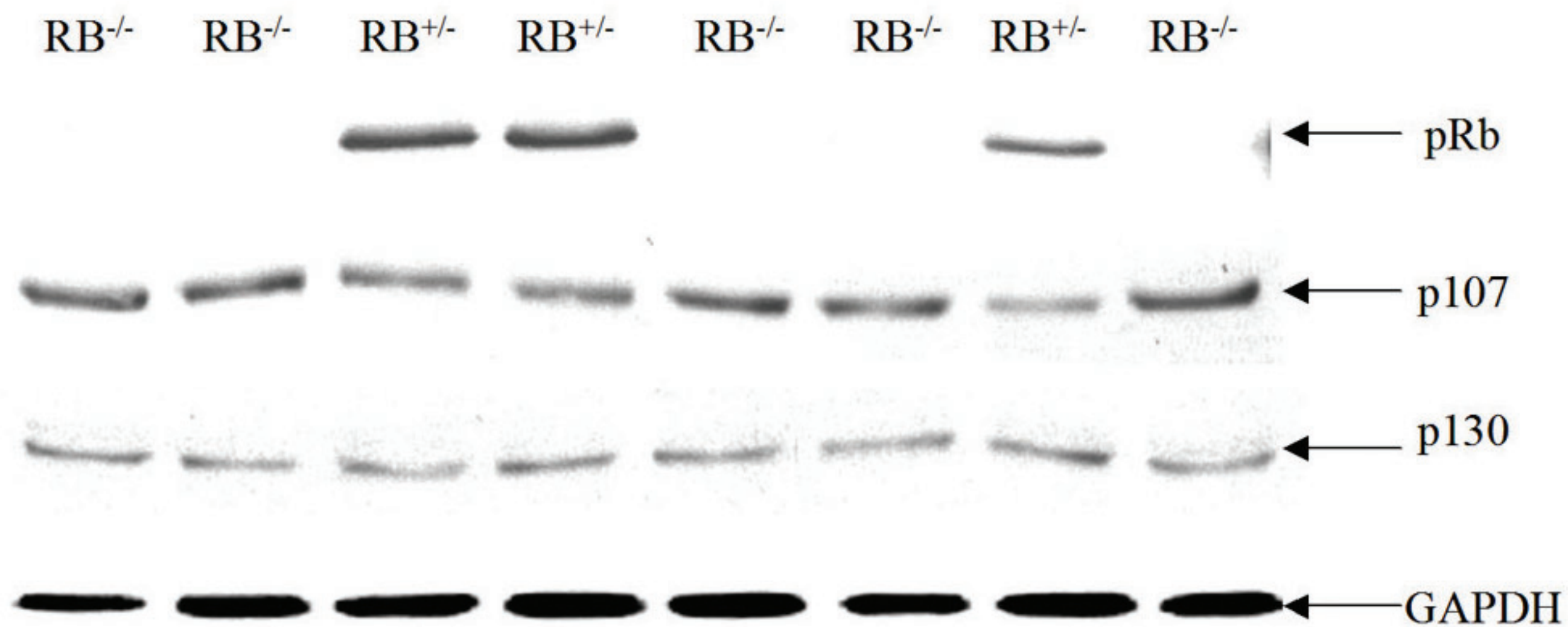

Supplement: Additional file 1 — Collegen1A1-specific Cre expression leads to pRb excision in vivo. (A) Cre-mediated recombination of "floxed" RB was detected by PCR analysis on intestine DNA isolated from E17.5 embryos (left). Cre transgene expression was assessed by PCR (right) (B) The expression levels of pRb family members: pRb, p107, and p130 in intestine derived from E17.5 embryos were detected by immunoblot analysis. GAPDH was used as a control. [file 1471-213X-7-6-S1.pdf]

A

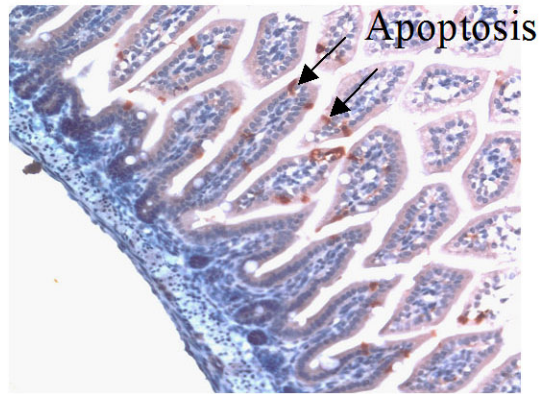

B

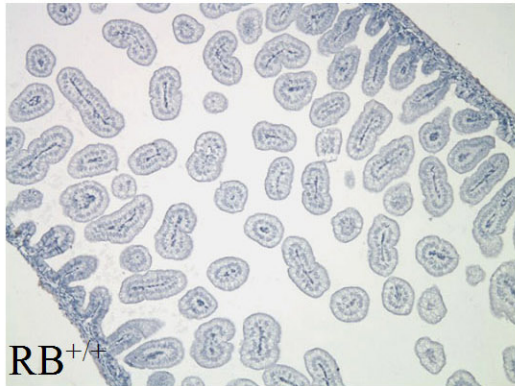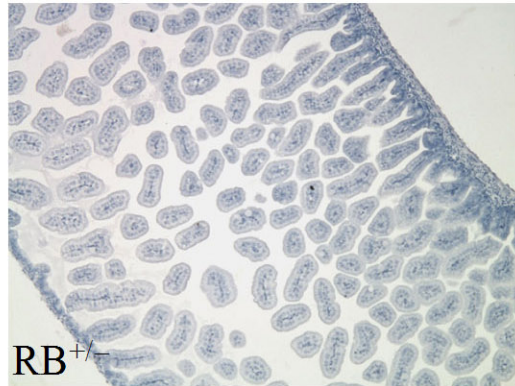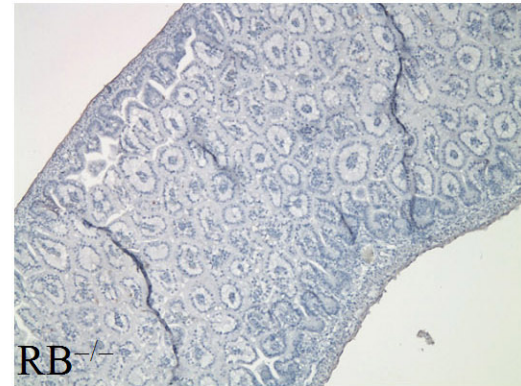

Supplement: Additional file 2 — Ablation of pRb has no effect on apoptosis. Apoptosis was detected with an antibody that recognizes cleaved caspase-3. Paraffin embedded intestine sections were derived from 3-week adult mice (A) and RB+/+, RB+/- and RB-/- newborn pups (B). Several apoptotic cells were detected on the tip of villi in adult mice, whereas different genotypes of RB newborn mice do not show apoptotic figures at this developmental stage. [file 1471-213X-7-6-S2.pdf]

A

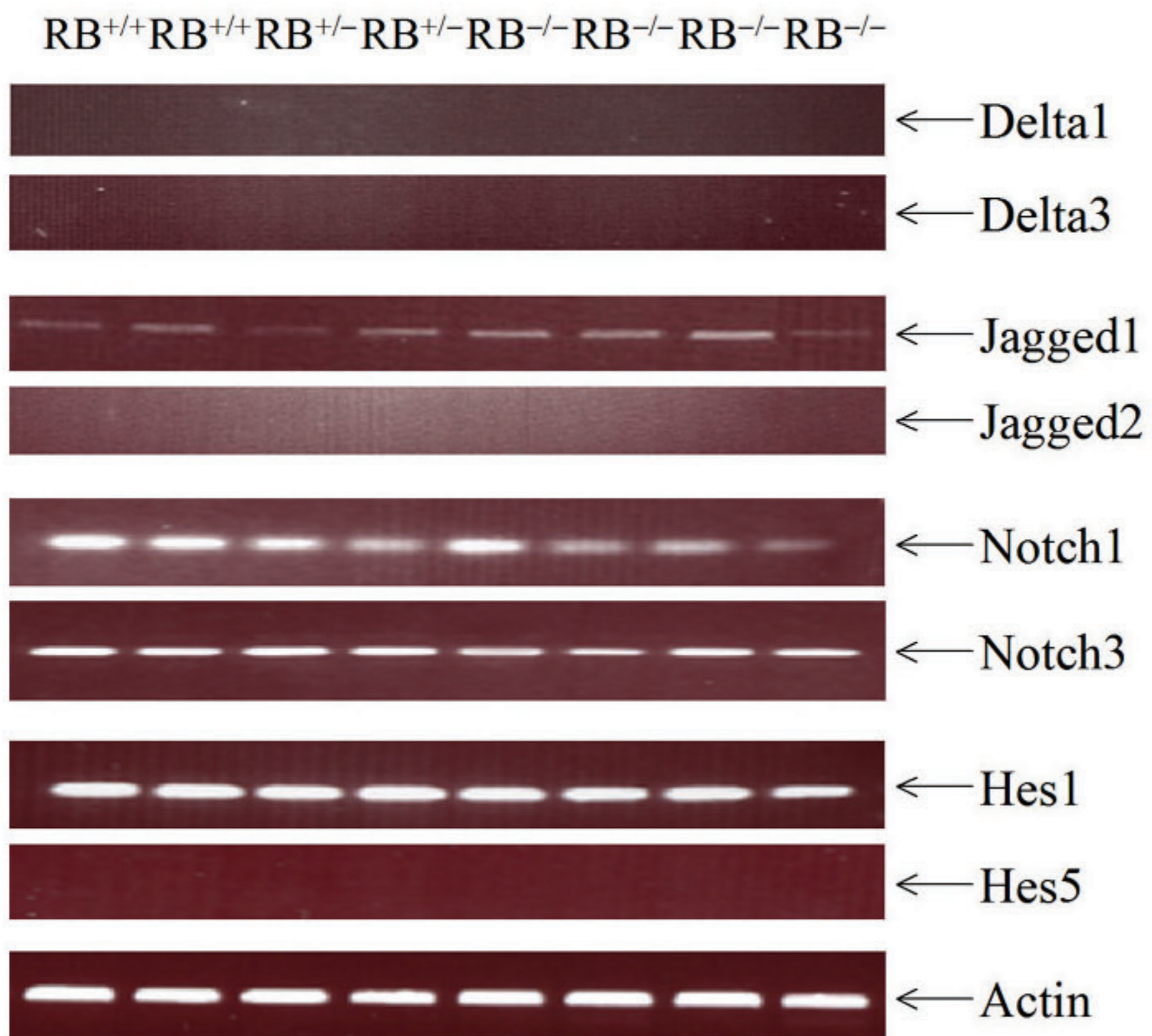

B

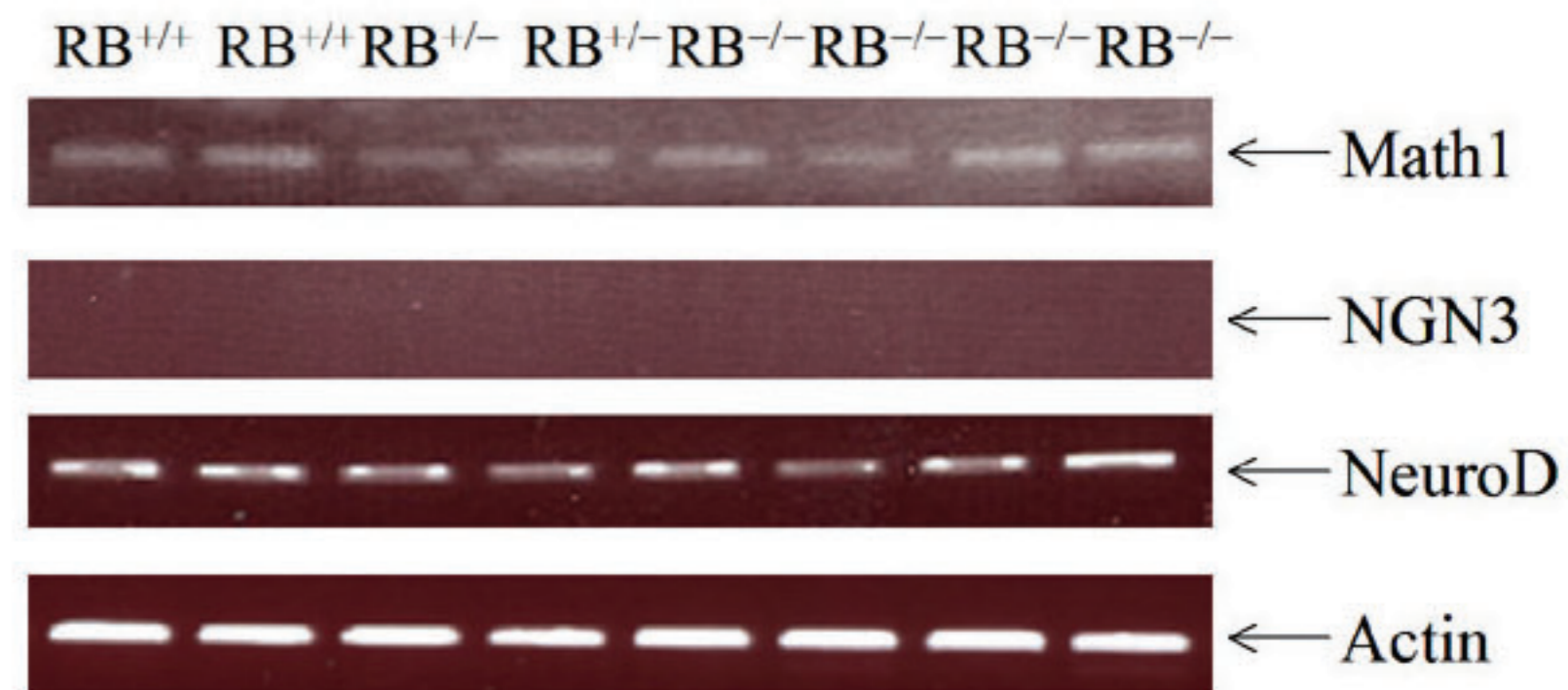

Supplement: Additional file 3 — Notch/Math pathway has little effect on the abnormal intestine with loss of pRb. RT-PCR amplification from P0 intestines derived from RB+/+, RB+/- and RB-/- newborn pups were performed to detect the expression levels of Delta1, Delta3, Jagged1, Jagged2, Notch1, Notch3, Hes1, Hes3 and actin used as a control (A) as well as Math1, Neurogenin3, and NeuroD (B). [file 1471-213X-7-6-S3.pdf]

A

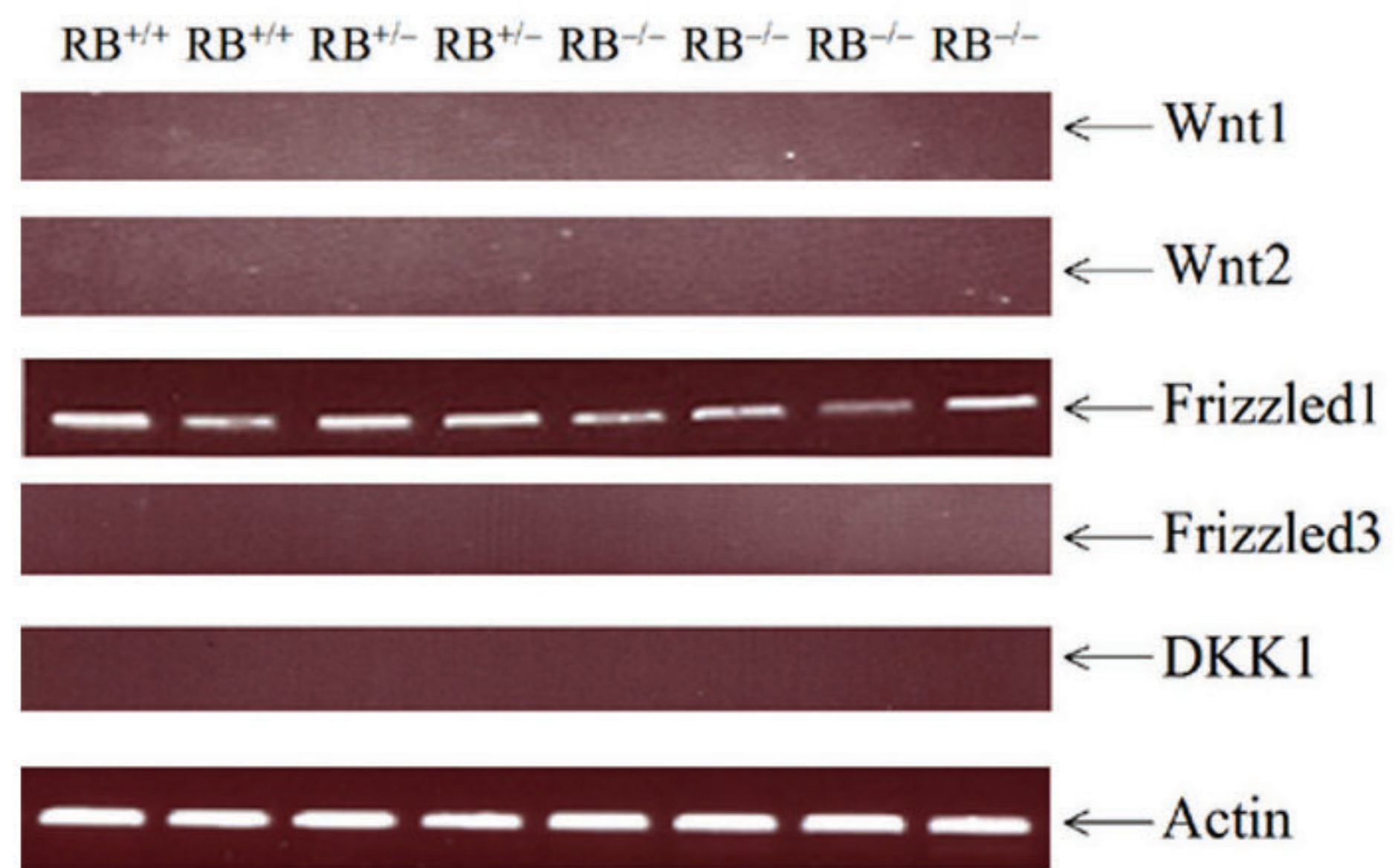

B

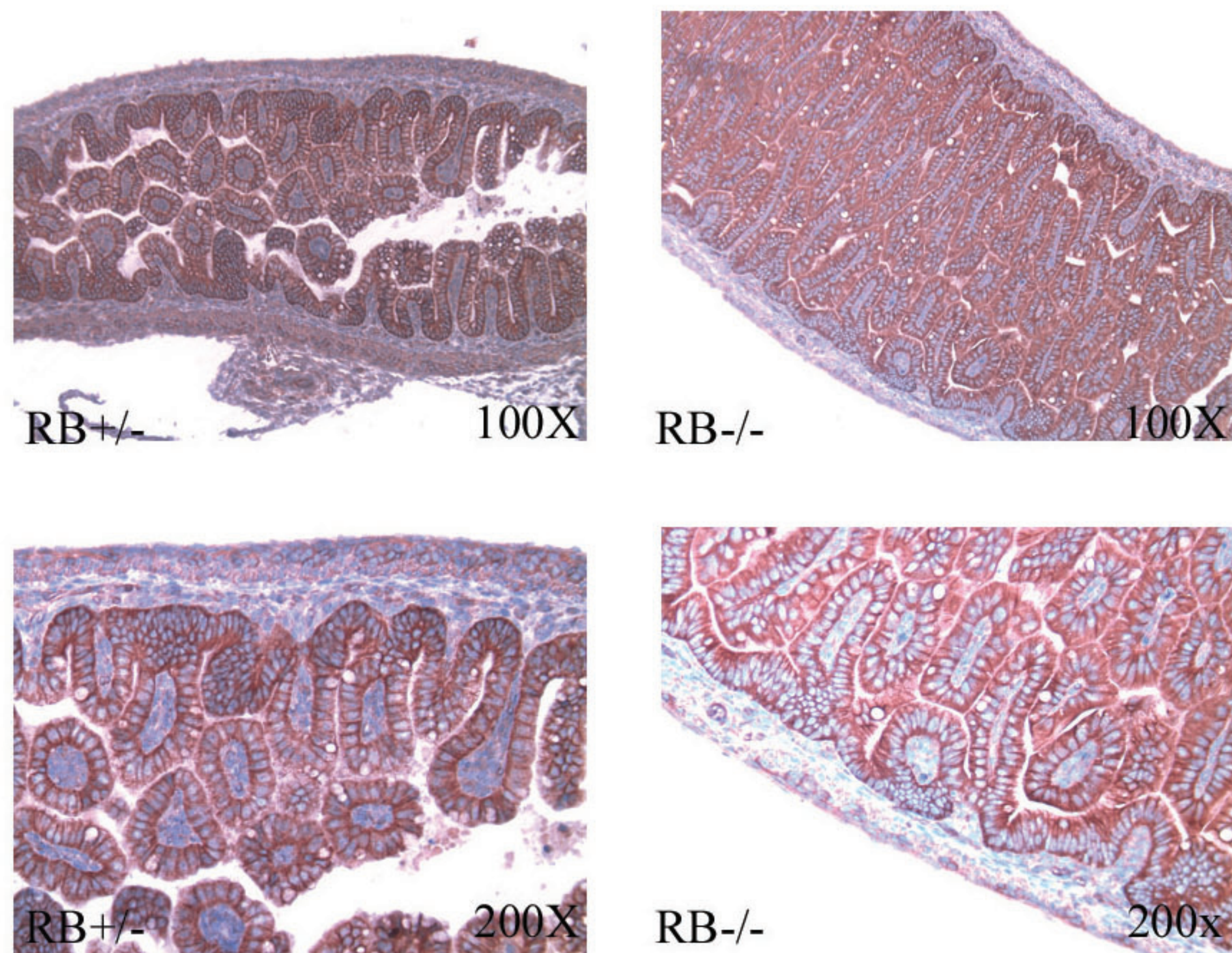

C

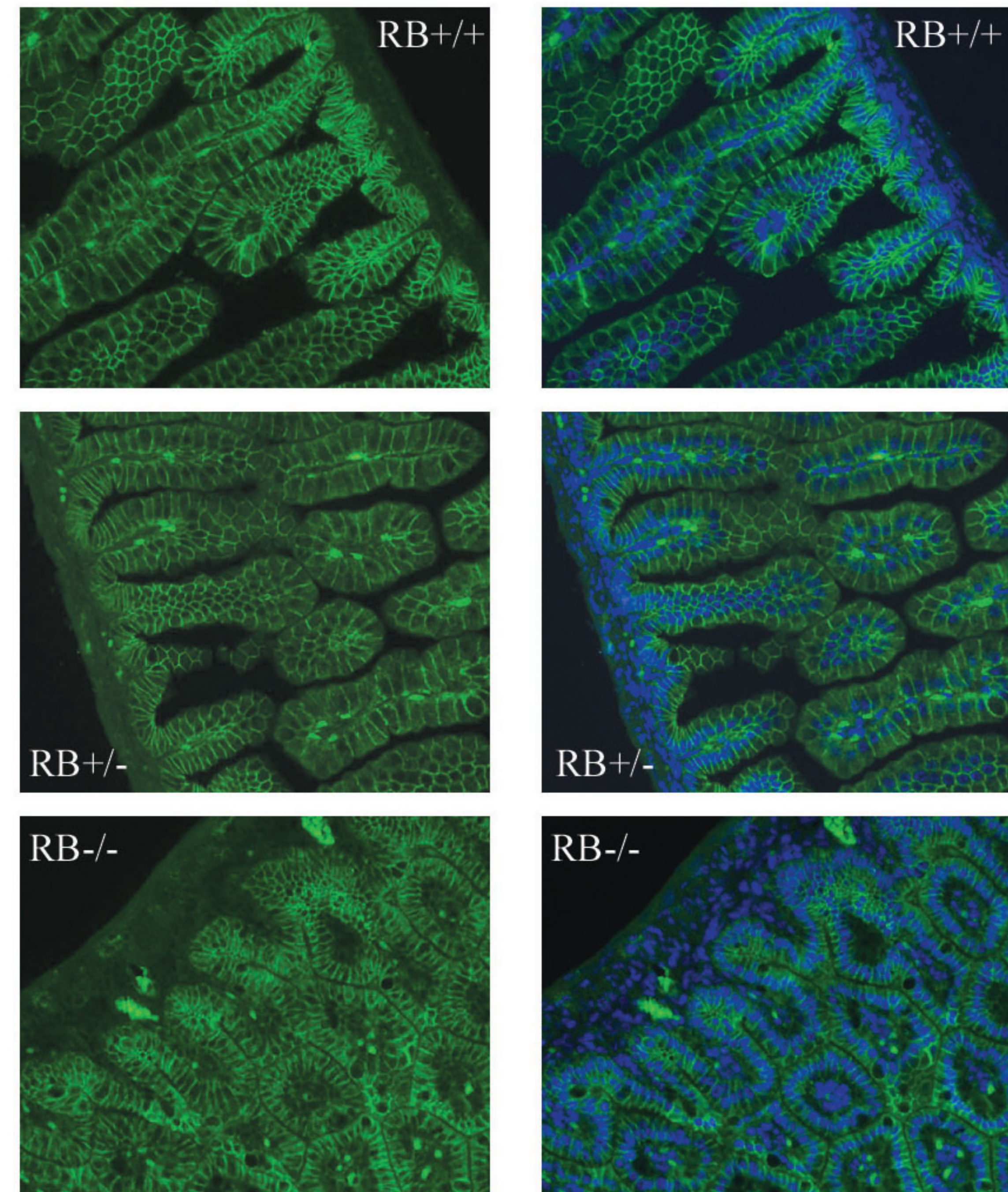

D

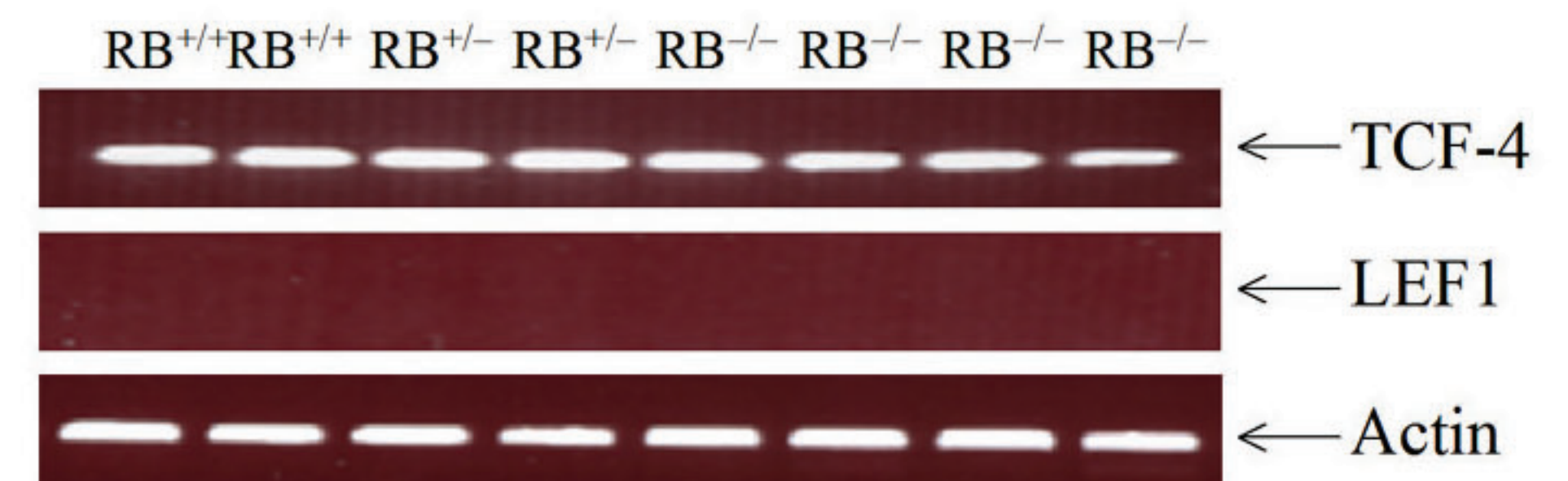

Supplement: Additional file 4 — Wnt signal does not contribute to the defect of intestine in the absence of pRb. Intestines derived from RB+/+, RB+/- and RB-/- newborn pups were subjected to RT-PCR amplification for the detection of Wnt1, Wnt2, Frizzled1, Frizzled3, DKK and actin used as a control (A) as well as TCF-4 and LEF1 (D). The embryonic intestine sections (E17.5) derived from RB+/- (left) or RB-/- (right) mice were stained by IHC with β-catenin (B). Representative sections from RB+/+, RB+/- and RB-/- newborn pups were stained with immunofluoresence by using anti- β-catenin (Green) and DAPI (Blue) (C). [file 1471-213X-7-6-S4.pdf]
